# Residential green space mitigates genetic susceptibility to incident irritable bowel syndrome in a prospective cohort of 376 749 individuals

**DOI:** 10.7189/jogh.16.04169

**Published:** 2026-06-12

**Authors:** Zhifu Kou, Rui Li, Jingjie Wang, Zhongjun Shao, Jin Feng, Mengna Wei, Ting Fu, Lan Chen, Guzhengyue Zheng, Hualiang Lin, Kun Liu

**Affiliations:** 1 Department of Gastroenterology, Tangdu Hospital, The Fourth Military Medical University, Xi’an, China; 2 The Ministry of Education Key Lab of Hazard Assessment and Control in Special Operational Environment, The Shaanxi Provincial Key Laboratory of Environmental Health Hazard Assessment and Protection, The Shaanxi Provincial Key Laboratory of Free Radical Biology and Medicine, Department of Epidemiology, School of Public Health, The Fourth Military Medical University, Xi’an, China; 3 Department of Epidemiology, School of Public Health, Sun Yat-sen University, Guangzhou, China; 4 Department of Remote Sensing and Information Engineering, Wuhan University, Wuhan, China; 5 Department of Epidemiology, School of Medicine, Xizang Minzu University, Xianyang, China

## Abstract

**Background:**

Environmental exposures may influence irritable bowel syndrome (IBS) pathogenesis, but the interplay between genetic risk, residential green/blue space, and IBS incidence remains unexplored.

**Methods:**

We aimed to study the association between green/blue space and incident IBS, and to assess effect modification by genetic susceptibility. The study included 376 749 UK Biobank participants at baseline. Residential green space was measured using the normalised difference vegetation index (NDVI), and blue space was defined by distance to the nearest water bodies. For time-to-event analyses, we used Cox proportional hazards models, polygenic risk scores (PRS), and mediation analysis to test gene-environment interactions. Results are presented as hazard ratios (HRs) with 95% confidence intervals (95% CIs).

**Results:**

During a follow-up of 11.73 years, 7091 incident IBS cases were documented. Higher residential green space exposure was associated with reduced risk of incident IBS. Specifically, within a 1000 m buffer, each interquartile range (IQR) increase in NDVI corresponded to a 5% lower IBS risk (HR = 0.95; 95% CI = 0.91–0.98), with consistent effects observed at 800 m and 500 m buffers. Conversely, a greater distance to water bodies was associated with reduced IBS risk. Stratification by PRS revealed a significant interaction: increased green space (1000 m buffer) reduced IBS risk by 8% exclusively among high-genetic-risk individuals (HR = 0.92; 95% CI = 0.88–0.96), whereas no interaction was observed for blue space. Moreover, physical activity mediated the association between green space and IBS.

**Conclusions:**

Increased residential green space exposure is associated with reduced IBS incidence, particularly among individuals with high genetic susceptibility. These findings underscore the importance of urban greening as a public health strategy to mitigate IBS risk, highlighting the potential for targeted environmental interventions to offset genetic predispositions.

Irritable bowel syndrome (IBS) is a chronic functional bowel disorder characterised by recurrent abdominal pain or discomfort associated with defecation or linked to changes in stool frequency/form, without identifiable structural pathology [1]. Globally affecting 3.80% of adults (Rome IV criteria), IBS impacts approximately 266 million individuals and imposes substantial socioeconomic burdens [2]. In the USA alone, it accounts for 3.1 million annual outpatient visits, with healthcare costs exceeding USD 20 billion [3]. Critically, the disorder’s heterogeneous manifestations suggest multifactorial origins involving gene-environment interactions [4].

Environmental factors are increasingly recognised as key contributors to IBS pathogenesis. Robust associations exist with discrete exposures, including air pollutants, microbial agents, and early-life factors such as pet ownership [5]. However, current evidence predominantly examines isolated risk factors, overlooking the synergistic effects of integrated environmental indicators. Residential green and blue spaces represent such composite measures, theoretically conferring multifaceted benefits through enhanced microbial diversity, stress reduction, and physical activity promotion [6,7]. Despite compelling mechanistic hypotheses, epidemiological evidence linking these holistic exposures to IBS risk remains limited and methodologically constrained.

A pivotal unexplored dimension is gene-environment interaction in IBS incidence. IBS-associated variants may fundamentally alter biological responses to environmental exposures [8]. Individuals with high polygenic risk could experience differential outcomes from green/blue space interventions – ranging from amplified benefits to unexpected adverse effects. However, large-scale evidence quantifying the effect modification of the environment by genetic susceptibility on IBS remains lacking, particularly regarding environmental interventions.

To address these critical gaps, we leveraged the UK Biobank (UKB) cohort to examine associations between residential green/blue space exposure and incident IBS and to evaluate potential effect modification by genetic susceptibility, quantified using polygenic risk scores (PRS). With this study, we aimed to inform urban planning strategies by clarifying the role of green and blue spaces in mitigating IBS risk.

## METHODS

### Study design and population

We obtained data from the UKB, a prospective cohort study that recruited 502 411 participants aged 37–73 years across the UK between 2006 and 2010. We adhered to GRABDROP guidelines and the STROBE checklist for observational studies (Appendix S1 and S2 in the **Online Supplementary Document**). All participants provided written informed consent, and ethical approval was granted by the UK North West Research Ethics Committee [9]. After excluding 25 542 baseline IBS cases and 100 120 individuals with incomplete covariate data, 376 749 participants were retained. For PRS derivation, we restricted analysis to individuals of European ancestry, which reflects the predominant genome-wide association studies (GWAS) population and excluded those with heterozygosity outliers, high missing genotype rates (>5%), genetic admixture and close relatedness (kinship coefficient >0.088) [10]. The final analytical cohort for PRS derivation comprised 356 712 individuals (Figure S1 in the **Online Supplementary Document**).

### Exposure assessment

**Figure 1:**
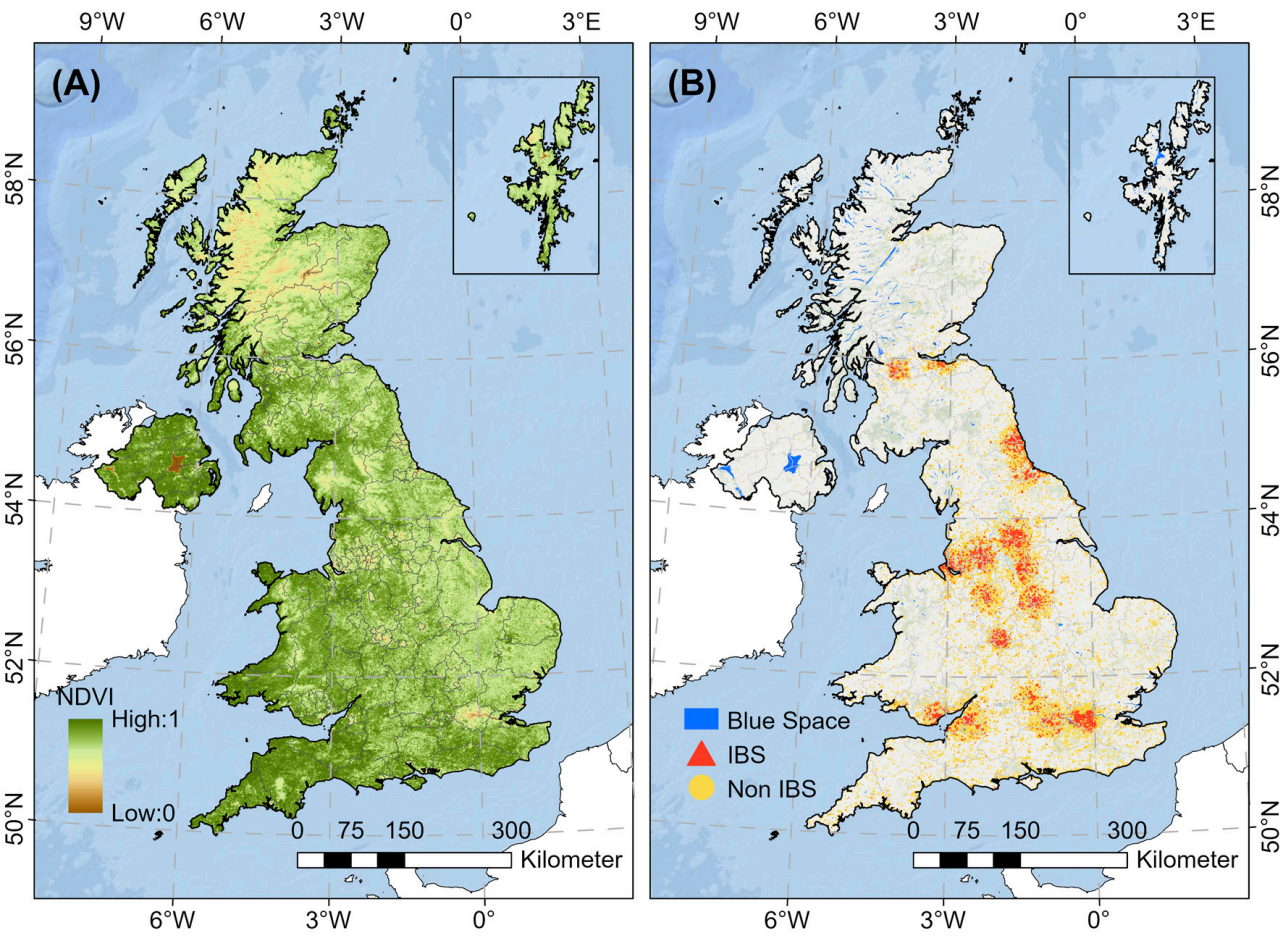
Spatial distribution of green and blue spaces in the UK. **Panel A.** Spatial distribution of NDVI in the UK. **Panel B.** Spatial distribution of blue space in the UK. IBS – irritable bowel syndrome, NDVI – normalised difference vegetation index.

To explore potential associations between IBS and residential green and blue spaces, we collected data on the normalised difference vegetation index (NDVI) and on blue spaces, which were then processed. NDVI, a proxy derived from land-surface reflectance in the visible and near-infrared portions of the spectrum, was used to measure exposure to green space in residential environments [11]. The NDVI data were derived from the 300 m resolution raster map of ‘Worldwide land cover mapping’ produced by the European Space Agency. Exposure to blue space was defined as proximity to inland water bodies (*e.g.* rivers, lakes, reservoirs). To analytically distinguish water bodies of different perceptual scales, the raster data were resampled to 50 m, 100 m, and 200 m resolutions using majority aggregation. Water bodies discernible at these respective resolutions were operationally classified as small, medium, and large. Residential exposure to blue space was quantified as the Euclidean distance from dwellings to the nearest water body within each category, using hydrographic data from the Joint Research Centre [12]. In addition, we used Esri’s ArcGIS, version 10.8.2 (Environmental Systems Research Institute, Redlands, California, USA) to create maps of green and blue spaces (**Figure 1**, Panels A and B).

### Outcome ascertainment

IBS cases and diagnosis dates were identified by linking hospital admissions, primary care, and self-reported data. Cases were defined as participants with either a primary or secondary International Statistical Classification of Diseases (ICD-9/ICD-10) diagnosis code for IBS (564.1 or K58) in hospital records, or whose symptoms met the Rome IV criteria for IBS based on questionnaire data. The follow-up for each participant was calculated from the date of the first assessment until the first date of new-onset IBS diagnosis, death, loss to follow-up, or the end of follow-up, whichever came first.

### Covariates

According to the availability of data, a large number of covariates associated with green and blue spaces and/or IBS were taken into the analysis, including age, sex (male or female), ethnicity (White, Asian, African descent, mixed race, other, or undeclared), residence (urban or rural), educational level (any school degree, college education, vocational qualification or other), income (<GBP 18 000, GBP 18 000–31 000, GBP 31 000–52 000, GBP 52 000–100 000,≥GBP 100 000, or unknown), body mass index (BMI) categorised as underweight <18.50 kg/m^2^ normal 18.50–24.90 kg/m^2^, overweight 25.00–29.90 kg/m^2^, or obese ≥30.00 kg/m^2^. Smoking status was classified as never smoker, former smoker, current smoker, or unknown; alcohol intake as heavy, moderate, occasional, or never; and physical activity level as low, moderate, or high.

### Polygenic risk scores (PRS)

We conducted PRS to investigate the effect of genetic factors. Six IBS-related single-nucleotide polymorphisms (SNPs) were obtained from the most recent GWAS [13] (Table S1 in the **Online Supplementary Document****)**. Each SNP was recorded as 0, 1, or 2, depending on the number of risk alleles. According to a previous study [14], genetic risk scores were calculated using the following equation:

IBS – PRS = (*β*_1_ × SNP_1_ + *β*_2_ × SNP_2_ + ... + *β*_6_ × SNP_6_) × (sum of *β* coefficients)

where the *β* coefficient is equal to the natural logarithm of the odds ratio of each SNP. Based on the tertiles of the PRS distribution among non-IBS participants, we categorised the continuous score into an ordinal variable with three levels: low risk (PRS ≤ 5.11), intermediate risk (5.11 < PRS ≤ 6.67), and high risk (PRS > 6.67), where higher scores indicate a greater genetic predisposition to IBS.

### Statistical analysis

The normality of continuous variables was evaluated using quantile-quantile plots (Figure S2 in the **Online Supplementary Document**). Continuous variables were expressed as medians and interquartile ranges (IQRs), and categorical variables as n (%). The Wilcoxon rank-sum test and the χ^2^ test were used to compare continuous and categorical variables, respectively. Cox proportional hazards regression models were used to examine the associations between exposure to green and blue spaces and the incidence of IBS. The exposures were treated as continuous variables, and hazard ratios (HRs) with corresponding 95% confidence intervals (95% CIs) were calculated for each one-unit increase in the IQR. Participants were divided into four groups based on exposure to green or blue spaces, with the first quartile serving as the reference group. The covariates were incrementally added to create three models: model 1 was designed to allow baseline risks to differ by age, sex, and ethnicity; model 2 was further modified to account for residence, income, and education level; and model 3 was further adjusted with BMI, smoking status, alcohol intake, and physical activity.

To evaluate nonlinear exposure-response relationships between green/blue space and incident IBS, we applied restricted cubic spline (RCS) models with five knots positioned at the 5th, 25th, 50th, 75th, and 95th percentiles. We utilised the ‘CMAverse’ *R* package to perform causal mediation analysis using 1000 bootstrap resamples to estimate confidence intervals. The analysis estimated the pure natural direct effect (PNDE), total natural indirect effect (TNIE), and proportion mediated (PM) of environmental exposures on IBS incidence. Physical activity was examined as a mediator, and interaction terms were included in the model to evaluate potential effect modification. Subsequently, interaction analyses and stratified analyses were conducted to further investigate these associations. Sensitivity analyses were conducted to assess robustness: restricting analyses to non-movers during follow-up, multiple imputation of missing covariate data (BMI, income, education level, smoking habits, alcohol intake, and physical activity) using random forests, and further adjustment for additional clinical covariates (hypertension and diabetes).

All statistical analyses were conducted as two-tailed tests using *R*, version 4.3.2 (R Core Team, Vienna, Austria) and ArcGIS, version 10.8 (Environmental Systems Research Institute, Redlands, California, USA). Two-sided tests with a *P*-value <0.05 were used as the threshold for statistical significance.

## RESULTS

### Demographic characteristics

During a follow-up of 11.73 years (4.30 million person-years), 7091 incident IBS cases were identified among 376 749 eligible baseline participants (**Table 1**). The median age of the cohort was 57 years (IQR = 50–63), with 182 943 males (48.56%). Most participants were White and resided in urban areas. In total, 250 302 participants (66.44%) were classified as being overweight or obese. IBS cases and non-cases differed in age, sex, BMI, and socioeconomic factors (income, education level, and alcohol intake). The baseline median NDVI was 0.71 (IQR = 0.56–0.81) in the 1000 m buffer, 0.73 (IQR = 0.57–0.82) in the 800 m buffer, and 0.76 (IQR = 0.59–0.86) in the 500 m buffer. Meanwhile, the median distances to large, medium, and small water bodies were 2.06 km (IQR = 1.20–3.23), 1.91 km (IQR = 1.13–3.02), and 1.83 km (IQR = 1.08–2.88), respectively.

**Table 1:** Baseline population characteristics according to incident IBS status*

Characteristics	All participants	IBS cases	Non-cases	*P*-value†
Participants	376 749	7091	369 658	
Age in years, MD (IQR)	57 (50–63)	57 (50–63)	57 (49–63)	0.002
Sex (male)	182 943 (48.56)	2223 (31.35)	180 720 (48.89)	<0.001
Ethnicity				0.625
*White*	356 712 (94.68)	6743 (95.09)	349 969 (94.67)	
*Asian*	7965 (2.11)	135 (1.90)	7830 (2.12)	
*Black*	5632 (1.49)	101 (1.42)	5531 (1.50)	
*Mixed*	2206 (0.59)	38 (0.54)	2168 (0.59)	
*Other*	3169 (0.84)	59 (0.83)	3110 (0.84)	
*Unknown*	1065 (0.28)	15 (0.21)	1050 (0.28)	
Overweight/obesity	250 302 (66.44)	4637 (65.40)	245 665 (66.45)	<0.001
Residence (urban)	319 785 (84.88)	6062 (85.49)	313 723 (84.87)	0.149
Income in GBP				<0.001
*<18 000*	70 272 (18.65)	1685 (23.76)	68 587 (18.55)	
*18 000–30 999*	83 374 (22.13)	1660 (23.41)	81 714 (22.11)	
*31 000–51 999*	89 174 (23.67)	1560 (22.00)	87 614 (23.70)	
*52 000–100 000*	72 896 (19.35)	1082 (15.26)	71 814 (19.43)	
*>100 000*	20 148 (5.35)	242 (3.41)	19 906 (5.38)	
*Unknown*	40 885 (10.85)	862 (12.16)	40 023 (10.83)	
Educational level				<0.001
*Any school degree*	143 659 (38.13)	2921 (41.19)	140 738 (38.07)	
*College education*	133 441 (35.42)	2120 (29.90)	131 321 (35.52)	
*Vocational qualification*	24 946 (6.62)	454 (6.40)	24 492 (6.63)	
*Other*	74 703 (19.83)	1596 (22.51)	73 107 (19.78)	
Smoking status				0.051
*Never*	205 895 (54.65)	3765 (53.10)	202 130 (54.68)	
*Previous*	131 578 (34.92)	2544 (35.88)	129 034 (34.91)	
*Current*	38 314 (10.17)	761 (10.72)	37 553 (10.16)	
*Unknown*	962 (0.26)	21 (0.30)	941 (0.25)	
Alcohol intake				<0.001
*Never*	27 414 (7.28)	26 704 (7.22)	710 (10.01)	
*Occasional*	80 638 (21.40)	78 792 (21.31)	1846 (26.03)	
*Moderate*	187 781 (49.84)	184 463 (49.90)	3318 (46.79)	
*Heavy*	80 916 (21.48)	79 699 (21.56)	1217 (17.16)	
Physical activity				<0.001
*Low*	70 446 (18.70)	1547 (21.82)	68 899 (18.64)	
*Moderate*	153 845 (40.83)	2858 (40.30)	150 987 (40.85)	
*High*	152 458 (40.47)	2686 (37.88)	149 772 (40.51)	
NDVI of green space in m, MD (IQR)				
*1000*	0.71 (0.56–0.81)	0.71 (0.56–0.80)	0.71 (0.56–0.80)	0.042
*800*	0.73 (0.57–0.82)	0.72 (0.57–0.82)	0.73 (0.57–0.82)	0.057
*500*	0.76 (0.59–0.86)	0.76 (0.59–0.85)	0.76 (0.59–0.86)	0.135
Distance to water bodies, MD (IQR)				
*Large water bodies*	2.06 (1.20–3.23)	1.99 (1.19–3.15)	2.06 (1.20–3.23)	0.010
*Medium water bodies*	1.91 (1.13–3.02)	1.87 (1.13–2.93)	1.92 (1.12–3.02)	0.006
*Small water bodies*	1.83 (1.08–2.88)	1.77 (1.07–2.74)	1.83 (1.08–2.88)	0.002

IBS – irritable bowel syndrome, IQR – interquartile range, MD – median, NDVI – normalised difference vegetation index

*Presented as n (%) unless specified otherwise.

†Continuous variables were compared using the Wilcoxon rank-sum test, and categorical variables using the χ^2^ test.

### Association of residential green and blue spaces with IBS incidence

Long-term residential green space exposure (NDVI) was associated with reduced IBS incidence (**Table 2**). In multivariable-adjusted models (model 3), within the 1000 m buffer, each IQR increase in NDVI was associated with a 5% lower IBS risk (HR = 0.95; 95% CI = 0.91–0.98). Compared with the lowest NDVI quartile (Q1), the HRs for developing IBS in the third (Q3) quartile were 0.91 (95% CI  = 0.85–0.98), and in the fourth (Q4) quartile were 0.90 (95% CI  = 0.83–0.96). Inverse associations per IQR increase were also observed for the 800 m (HR = 0.95; 95% CI = 0.92–0.98) and 500 m buffers (HR = 0.97; 95% CI = 0.93–0.99). However, when analysed by quartiles, a reduced risk was only evident within the 1000 m buffer. Associations in model 2 were consistent for significant findings, whereas model 1 generally showed weaker or non-significant associations.

**Table 2:** Associations between green space and the incidence of IBS

Green space		Model 1*		Model 2*		Model 3*	
	**Cases, n (%)†**	**HR (95% CI)**	***P-*value**	**HR (95% CI)**	***P-*value**	**HR (95% CI)**	***P-*value**
NDVI 1000 m buffer							
*Per IQR increment*	7091	0.98 (0.95–1.01)	0.105	0.95 (0.92–0.98)	0.004	0.95 (0.91–0.98)	0.001
*Q1*	1813 (25.57)	ref		ref		ref	
*Q2*	1813 (25.57)	0.99 (0.93–1.06)	0.775	0.96 (0.90–1.03)	0.268	0.96 (0.90–1.03)	0.238
*Q3*	1756 (24.76)	0.96 (0.89–1.02)	0.175	0.92 (0.85–0.98)	0.016	0.91 (0.85–0.98)	0.010
*Q4*	1709 (24.10)	0.94 (0.88–1.01)	0.071	0.91 (0.84–0.98)	0.010	0.90 (0.83–0.96)	0.003
NDVI 800 m buffer							
*Per IQR increment*	7091	0.96 (0.92–0.99)	0.011	0.96 (0.92–0.99)	0.011	0.95 (0.92–0.98)	0.004
*Q1*	1776 (25.05)	ref		ref		ref	
*Q2*	1847 (26.05)	1.00 (0.94–1.07)	0.956	1.00 (0.94–1.07)	0.956	1.00 (0.93–1.07)	0.963
*Q3*	1747 (24.63)	0.94 (0.87–1.01)	0.071	0.94 (0.87–1.01)	0.071	0.93 (0.86–0.99)	0.046
*Q4*	1721 (24.27)	0.94 (0.88–1.02)	0.122	0.94 (0.88–1.02)	0.122	0.93 (0.87–1.00)	0.063
NDVI 500 m buffer							
*Per IQR increment*	7091	0.97 (0.94–1.00)	0.081	0.97 (0.94–1.00)	0.081	0.97 (0.93–0.99)	0.045
*Q1*	1743 (24.58)	ref		ref		ref	
*Q2*	1860 (26.23)	1.03 (0.96–1.10)	0.442	1.03 (0.96–1.10)	0.442	1.02 (0.96–1.10)	0.502
*Q3*	3405 (48.02)	1.00 (0.93–1.08)	0.925	1.00 (0.93–1.08)	0.925	1.00 (0.93–1.07)	0.931
*Q4*	83 (1.17)	0.94 (0.88–1.02)	0.128	0.94 (0.88–1.02)	0.128	0.94 (0.87–1.01)	0.071

CI – confidence interval, HR – hazard ratio, IBS – irritable bowel syndrome, IQR – interquartile range, NDVI – normalised difference vegetation index, ref – reference, Q – quantile

*Model 1 adjusted for age, sex, and ethnicity. Model 2 additionally adjusted for residence, income, and educational level. Model 3 was further adjusted for BMI, smoking status, alcohol intake, and physical activity.

†n represents the number of cases, with the percentage in parentheses indicating the proportion of cases within the total cases (N = 7 091).

A negative relationship was observed between the distance to the nearest water bodies and the incidence of IBS (Table S2 in the **Online Supplementary Document**). The incidence of IBS was associated with distance to the nearest water bodies when distance was considered as a continuous variable. In model 3, each IQR increase in distance to water bodies was associated with a 3.0% reduction in IBS incidence for large/medium water bodies (HR = 0.97; 95% CI = 0.94–0.99) and a 4.0% reduction for small water bodies (HR = 0.96; 95% CI = 0.93–0.99).

**Figure 2:**
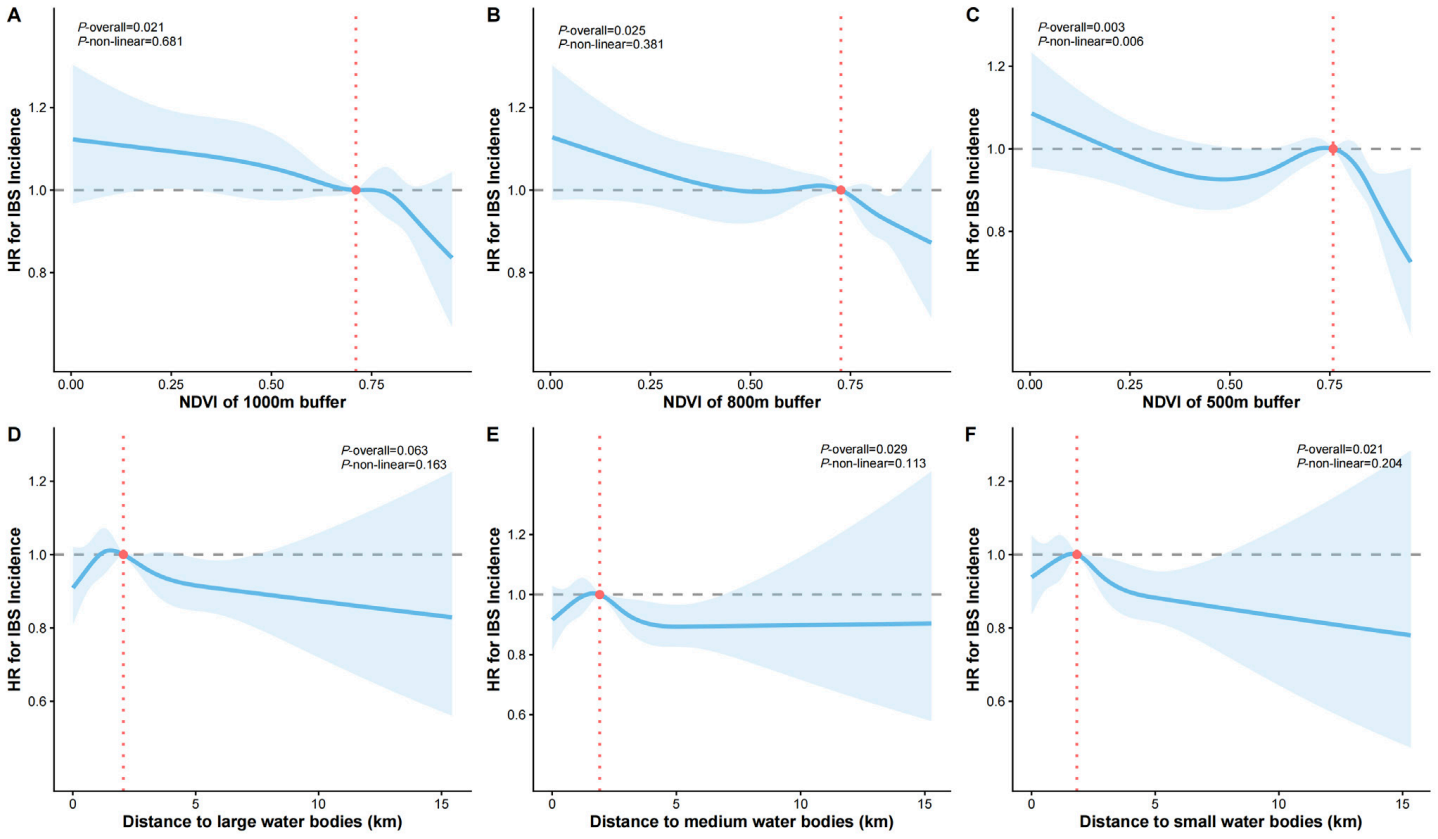
RCS for the relationship between green/blue space and IBS incidence. **Panel A.** NDVI within a 1000m buffer. **Panel B.** NDVI within an 800m buffer. **Panel C.** NDVI within a 500m buffer. **Panel D.** Distance to the nearest large water bodies. **Panel E.** Distance to the nearest medium water bodies. **Panel F.** Distance to the nearest small water bodies. HR – hazard ratio, IBS – irritable bowel syndrome, NDVI – normalised difference vegetation index, RCS – restricted cubic spline.

The RCS analyses revealed complex, predominantly nonlinear associations between environmental factors and the HR for IBS incidence. Regarding green space, decreasing trends in IBS risk were observed across different buffer zones after adjusting for potential confounders (**Figure 2**, Panels A and B). Notably, the relationship between the 500 m buffer and IBS incidence was nonlinear (*P*-overall = 0.003, *P-*nonliner = 0.006). Increased distance to water bodies was generally associated with lower IBS incidence. For large water bodies, the overall association was not significant (*P*-overall = 0.063). However, significant overall associations for medium (*P*-overall = 0.029) and small water bodies (*P*-overall = 0.021) (**Figure 2**, Panels C–F). No significant nonlinearity was found for the distance associations of water bodies.

### PRS-stratified analysis of residential environment and IBS risk

The interaction between residential green/blue space and genetic predisposition to IBS was assessed through PRS stratification. For green space exposure, high-PRS individuals had a lower incidence of IBS with increased residential greenness (**Table 3**). Specifically, within the NDVI 1000 m buffer, each IQR increment in green space was associated with an 8.0% reduction in IBS risk exclusively in the high-PRS group (HR = 0.92; 95% CI = 0.87–0.96), whereas no significant association was observed in low-PRS individuals (HR = 0.97; 95% CI = 0.92–1.02). Furthermore, compared to Q1, Q3, and Q4 conferenced 18.0% (HR = 0.82; 95% CI = 0.75–0.91) and 14.0% (HR = 0.86; 95% CI = 0.77–0.95) risk reductions, respectively. Smaller buffers (800 m and 500 m) exhibited attenuated protective effects among high-PRS individuals, with significant per-IQR trends but inconsistent quartile-based associations. In contrast, no significant association between blue space exposure and IBS risk was detected in PRS-stratified analysis (Table S3 in the **Online Supplementary Document**).

**Table 3:** Association between green space and IBS incidence stratified by PRS*

Green space	Low genetic risk		High genetic risk	
	**HR (95% CI)**	***P-*value**	**HR (95% CI)**	***P-*value**
NDVI 1000 m buffer				
*Per IQR increment*	0.97 (0.92–1.02)	0.265	0.92 (0.87–0.96)	<0.001
*Q1*	ref		ref	
*Q2*	0.93 (0.84–1.03)	0.185	0.95 (0.87–1.05)	0.326
*Q3*	0.98 (0.88–1.09)	0.663	0.82 (0.75–0.91)	<0.001
*Q4*	0.91 (0.81–1.01)	0.081	0.86 (0.77–0.95)	0.003
NDVI 800 m buffer				
*Per IQR increment*	0.97 (0.92–1.02)	0.272	0.93 (0.88–0.97)	0.001
*Q1*	ref		ref	
*Q2*	0.97 (0.87–1.08)	0.584	0.98 (0.89–1.08)	0.684
*Q3*	0.99 (0.89–1.10)	0.862	0.9 (0.81–0.99)	0.032
*Q4*	0.95 (0.85–1.06)	0.367	0.91 (0.82–1.00)	0.062
NDVI 500 m buffer				
*Per IQR increment*	0.99 (0.93–1.04)	0.581	0.94 (0.89–0.98)	0.008
*Q1*	ref		ref	
*Q2*	1.02 (0.92–1.13)	0.756	0.97 (0.88–1.07)	0.511
*Q3*	1.06 (0.96–1.19)	0.250	0.94 (0.85–1.04)	0.238
*Q4*	0.92 (0.82–1.03)	0.148	0.91 (0.82–1.00)	0.056

CI – confidence interval, HR – hazard ratio, IBS – irritable bowel syndrome, IQR – interquartile range, NDVI – normalised difference vegetation index, ref – reference, Q – quantile

*Cox proportional hazard model adjusted by age, sex, ethnicity, residence, income, educational level, smoking habit, alcohol intake, BMI, and physical activity.

### Physical activity mediates the protective effect of green space against IBS

**Figure 3:**
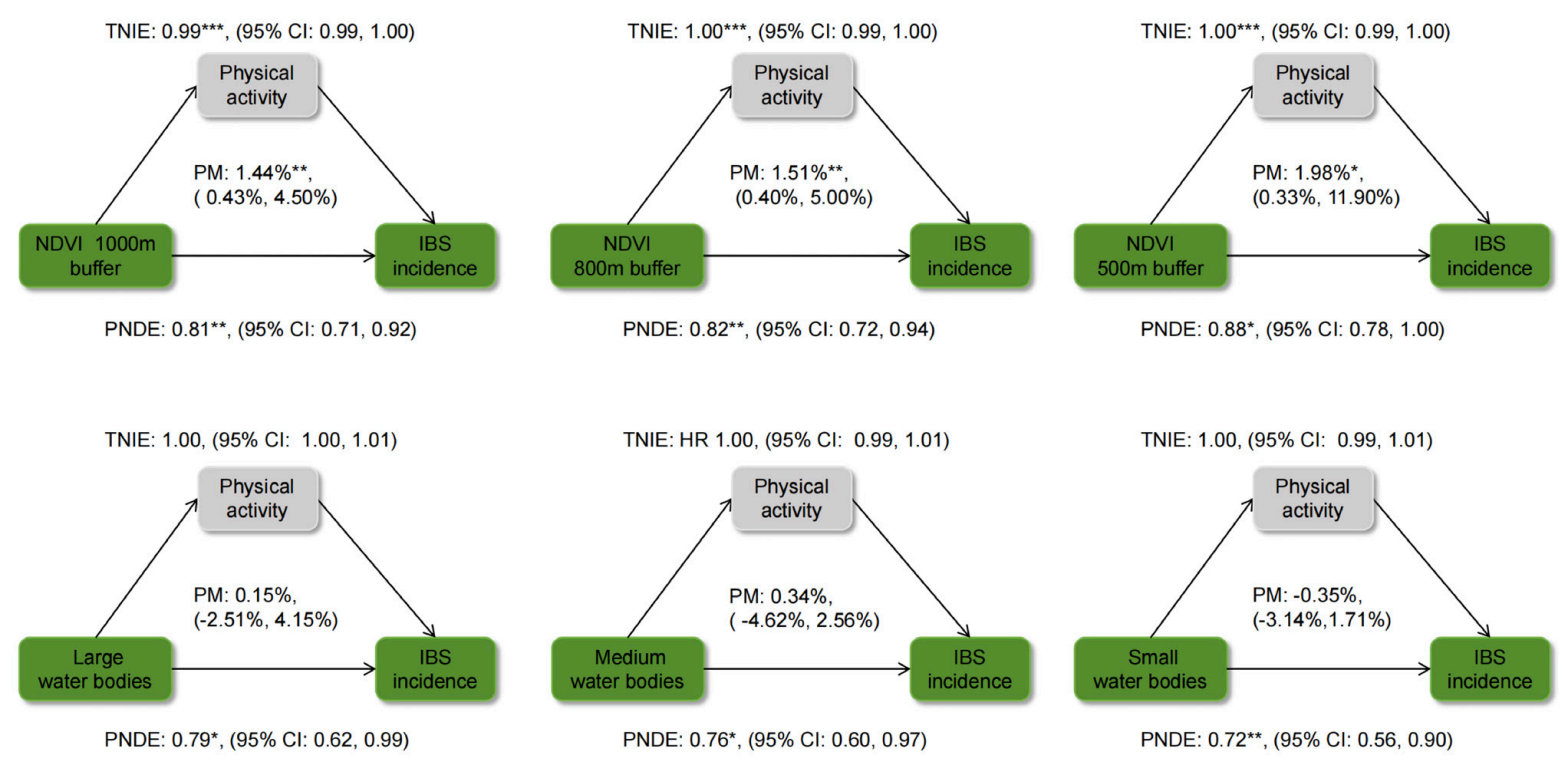
Mediating effects of physical activity on the associations between green/blue space exposure and incident IBS risk. Models were adjusted for age, sex, ethnicity, area of residence, household income, educational attainment, smoking status, alcohol consumption and BMI. *P < 0.05. **P < 0.01. ***P < 0.001, IBS – irritable bowel syndrome, NDVI – normalised difference vegetation index, PM – proportion mediated, PNDE – pure natural direct effect, TNIE – total natural indirect effect.

Distinct mediation patterns of different environmental exposures on IBS incidence via physical activity (**Figure 3**). For higher greenness exposure, significant protective mediation by physical activity was observed across all buffer sizes (500 m, 800 m, 1000 m). This was evidenced by statistically significant TNIEs and PM estimates ranging from 1.44% to 1.98%. In contrast, physical activity did not significantly mediate the protective associations observed for distance to water bodies (large, medium, small), as indicated by non-significant TNIE estimates and PM estimates. Notably, robust protective direct effects (HRs of 0.72–0.88) were consistently observed across all environmental exposures, irrespective of green- or blue-space classification.

### Interaction, subgroup and sensitivity analyses

No significant multiplicative interactions were observed between green space and blue space. Based on the subgroup analyses, the patterns of association differed between green and blue spaces. Statistically significant protective effects for green space were observed among females and urban residents across all scales. However, no significant interactions were detected for any subgroup. For blue space, inverse associations were consistently observed among males (with significant sex-based interactions) and among urban residents and the low physical activity group across all scales (Figures S2 and S3 and Table S4 in the **Online Supplementary Document**). After multiple testing corrections and various sensitivity analyses (including non-mover stratification, imputation of missing covariate data, and the addition of covariates for hypertension and diabetes), the association between green space exposure and IBS incidence remained consistent. In contrast, the association with blue space exposure was less robust after adjusting for non-movers during follow-up, whereas other sensitivity analyses yielded consistent results (Tables S5–10 in the **Online Supplementary Document**).

## DISCUSSION

This pioneering study reveals significant associations between residential green/blue space and IBS incidence. Higher residential greenness and greater proximity to blue space were associated with a reduced risk of IBS. Crucially, green space reduced the risk of IBS in high-genetic-risk individuals, suggesting a gene-environment interaction in IBS pathogenesis. These findings suggest that urban greening is a promising preventive strategy against IBS.

Although our results suggest an association between green space and reduced IBS risk, direct epidemiological evidence linking green space exposure to IBS incidence remains limited. The observed association may operate through several plausible mechanisms. First, residential green space enhances biodiversity, increasing exposure to microbial diversity [15], which may counteract gut microbiota dysbiosis implicated in the pathogenesis of IBS. Second, visual and auditory stimuli from green environments may down-regulate hypothalamic-pituitary-adrenal axis activity, thereby reducing stress responses that exacerbate IBS [16]. Furthermore, our mediation analysis identifies physical activity as a partial mechanistic conduit against the development of IBS [17], consistent with established evidence of its protective effects. However, the modest mediation proportion (1.44–1.98%) indicates other significant pathways require further investigation.

Our analyses demonstrate an inverse dose-response relationship between residential green space exposure and the risk of IBS incidence, with spatial-scale dependence. This gradient was corroborated by multivariable models: per-IQR NDVI increases conferred progressively stronger protection with larger buffers – peak effect at 1000 m (HR = 0.95; 95%CI = 0.91–0.98) *vs.* attenuated effect at 500 m (HR = 0.97; 95%CI = 0.93–0.99). Critically, RCS analysis revealed significant nonlinearity exclusively at the 500 m buffer (*P*-nonlinear = 0.006), indicating distinct exposure-response dynamics for localised vegetation that necessitate mechanistic exploration of scale-dependent pathways.

Notably, our findings reveal a counterintuitive positive association between closer proximity to water bodies and elevated IBS incidence. We propose that this paradoxical pattern may reflect increased exposure to waterborne pathogens in contaminated water bodies – specifically, small artificial ones – potentially establishing a causal pathway to post-infectious IBS [18]. This interpretation aligns with contemporary concerns regarding water quality degradation stemming from industrialisation and urbanisation [19]. Furthermore, small water bodies typically exhibit limited self-purification capacity and consequently greater susceptibility to microbial contamination [20,21]. Critically, biological plausibility is demonstrated through established waterborne pathogen-post-infectious-IBS linkages [22], while meta-analyses confirm a 4-fold elevated IBS risk post-enteritis [23]. Furthermore, recreational use of nearby water bodies may increase exposure to contaminants, allergens, or pathogens, thereby raising the risk of IBS via the dual pathways of inducing gastrointestinal infections and/or disrupting gut homeostasis [24,25]. Finally, while blue space is generally considered restorative, certain types (*e.g.* those perceived as unsafe, odorous, or associated with flood risk) may conversely act as a psychosocial stressor, thereby exacerbating IBS symptoms via the gut-brain axis [26,27]

While the combined effects of genetics and environmental factors on IBS risk remain understudied, our study demonstrates that residential green space may reduce IBS incidence among individuals at high genetic risk. These findings suggest that epigenetic mechanisms may play a significant role in IBS pathogenesis [28]. Notably, this protective effect was most pronounced within a 1000 m buffer compared to other buffer sizes. However, we observed no association between green space exposure and IBS incidence in individuals with low genetic risk. This suggests that the beneficial association of green space with reduced IBS risk may be modified by genetic susceptibility, highlighting a potential gene-environment interaction. Furthermore, no significant association was found between proximity to blue space and IBS risk across all levels of genetic risk. Consequently, further investigation is needed into potential gene-environment interactions, specifically examining the effect of green space activities on physiological pathways.

Our study offers several key contributions. It represents the first prospective investigation of the associations between exposure to residential green/blue spaces and the incidence of IBS. We utilised high-precision NDVI data to quantify green space, enhancing the accuracy of exposure assessment. To delve into gene-environment interplay, PRS were used to examine how associations with green space vary among individuals with different genetic susceptibilities to IBS. Finally, preliminary exploration of potential mediating pathways was conducted to offer initial mechanistic insights.

However, several important limitations warrant careful consideration and temper the interpretation of our findings. First, the UKB cohort predominantly comprises individuals of Caucasian European ancestry, which significantly limits the generalisability of our results to diverse ethnic populations, where genetic backgrounds and environmental interactions may differ. Second, our study focused on middle-aged and older adults and lacked crucial data on environmental exposures during formative early-life stages (childhood and adolescence), which might be critical for IBS development. Third, the NDVI metric captures general greenness but not specific vegetation types, quality, or their temporal variation during follow-up. Therefore, our results pertain to baseline green space quantity rather than its qualitative or dynamic attributes. Fourthly, our blue space exposure metrics (focusing on proximity to inland waters) do not capture exposure to marine environments or qualitative aspects (*e.g.* water quality), and the ecological design precludes strong causal inference. Finally, the ecological study design, static exposure metrics, and potential residual confounding (*e.g.* air pollution); all warrant caution in interpreting the observed associations. Future interdisciplinary research is essential to explore the specific types and qualities of green/blue space, their mechanisms of action (including mediation pathways), and their impact across different life stages and populations.

## CONCLUSIONS

This large-scale prospective cohort study comprehensively examined the associations between residential green and blue spaces and the incidence of IBS. Our findings indicate that greater green space is associated with a reduced risk of IBS, highlighting the potential value of urban greening as a preventive strategy. These results suggest integrating green infrastructure into urban planning as a public health measure to mitigate IBS incidence, particularly in genetically susceptible populations.

## Additional material


Online Supplementary Document
