# Supplementary material for: Residential green space mitigates genetic susceptibility to incident irritable bowel syndrome in a prospective cohort of 376 749 individuals
Source: J Glob Health. 2026 Jun 12;16:04169. doi: 10.7189/jogh.16.04169 (PMC13261328; doi:10.7189/jogh.16.04169)
Supplement: Online Supplementary Document [file jogh-16-04169-s001.pdf]

Supplement to: Kou Z, Li R, Wang J, Shao Z, Feng J, Wei M, Fu T, Chen L, Zheng G, Lin H, Liu K. Residential green space mitigates genetic susceptibility to incident irritable bowel syndrome in a prospective cohort of 376 749 individuals. J Glob Health. 2026;16:04169.

Appendix S1. Outline of JoGH's Guidelines for Reporting Analyses of Big Data Repositories Open to the Public (GRABDROP) items

JoGH guideline item	Purpose	Response
1. Please list all papers published by each co-author in previous three years that were based on secondary analysis of a big data repository	Preventing publications from possible 'paper mills' and authorships granted without genuine contribution	A hypothetical intervention analysis for the effects of healthy dietary patterns on reducing major chronic diseases and mortality associated with air pollutant mixtures [2025, Jin Feng; 2025, Guzhengyue Zheng; 2025, Hualiang Lin]<br><br>Air pollution associated with cardiopulmonary disease and mortality among participants with preserved ratio impaired spirometry [2024, Guzhengyue Zheng; 2024, Hualiang Lin]<br><br>Air pollution metabolomic signatures and chronic respiratory diseases risk: A longitudinal study [2024, Hualiang Lin]<br><br>Air pollution, APOE genotype and risk of dementia among individuals with cardiovascular diseases: A population-based longitudinal study [2024, Hualiang Lin]<br><br>Air pollution, metabolic signatures, and the risk of idiopathic pulmonary fibrosis [2025, Jin Feng; 2025, Hualiang Lin]<br><br>Ambient air pollution exposure accelerates the occurrence of 78 non-communicable chronic diseases: an accelerated failure time analysis of a nationwide cohort [2026, Hualiang Lin]<br><br>Association of metabolic signatures of air pollution with MASLD: Observational and Mendelian randomization study [2025, Hualiang Lin]

Associations between air pollution and the risk of first admission and multiple readmissions for cardiovascular diseases [2024, Lan Chen; 2024, Hualiang Lin]

Associations between Changes in Exposure to Air Pollutants due to Relocation and the Incidence of 14 Major Disease Categories and All-Cause Mortality: A Natural Experiment Study [2025, Hualiang Lin]

Associations of air pollution and genetic risk and their interaction with risk of Alzheimer's disease: identification of risk loci and potential biological pathways [2026, Lan Chen; 2026, Hualiang Lin]

Associations of ambient air pollution with incidence and dynamic progression of atrial fibrillation [2024, Hualiang Lin]

Associations of Fish and Fish Oil Consumption With Incident Inflammatory Bowel Disease: A Population-Based Prospective Cohort Study [2024, Lan Chen; 2024, Hualiang Lin]

Associations of glycosylated hemoglobin, pre-diabetes, and type 2 diabetes with incident lung cancer: A large prospective cohort study [2024, Hualiang Lin]

Associations of lifetime exposure to fine particulate matter (PM2.5) and its constituents with increased risk and earlier occurrence of 14 site-specific cancers [2026, Lan Chen; 2026, Hualiang Lin]

Associations of MAFLD subtypes and air pollutants with multi-system morbidity and all-cause mortality: A prospective cohort study [2025, Lan Chen; 2025, Hualiang Lin]

Causal association between long-term exposure to air pollution and incident Parkinson's disease [2024, Lan Chen; 2024, Hualiang Lin]

Characterizing Metabolic Signatures of Air Pollution and Their Association With the Risk of Gout: A Population-Based Cohort Study [2025, Lan Chen; 2025, Hualiang Lin]

Characterizing metabolomic signatures related to coffee and tea consumption and their association with incidence and dynamic progression of type 2 diabetes: a multi-state analysis [2025, Guzhengyue Zheng; 2025, Hualiang Lin]

Differential associations of constituents of ambient fine particulate matter with 12 cardiovascular diseases: A prospective study [2026, Lan Chen; 2026, Hualiang Lin]

Early-life exposure to tobacco, inflammation, and risk of rheumatic diseases: a prospective cohort study [2025, Hualiang Lin]

Effect modification of dietary diversity on the association of air pollution with incidence, complications, and mortality of type 2 diabetes: Results from a large prospective cohort study [2024, Guzhengyue Zheng; 2024, Hualiang Lin]

Fresh fruit, dried fruit, raw vegetables, and cooked vegetables consumption associated with progression trajectory of type 2 diabetes: a multi-state analysis of a prospective cohort [2024, Guzhengyue Zheng; 2024, Hualiang Lin]

Healthy Lifestyle, Metabolic Signature, and Risk of Cardiovascular Diseases: A Population-Based Study [2025, Lan Chen; 2025, Hualiang Lin]

High Dietary Intake of Iron Might Be Harmful to Atrial Fibrillation and Modified by Genetic Diversity: A Prospective Cohort Study [2024, Hualiang Lin]

Impact of air pollution and multimorbidity on the risk of incident atrial fibrillation [2025, Hualiang Lin]

Interactive effects of physical activity and sarcopenia on incident ischemic heart disease: Results from a nation-wide cohort study [2024, Lan Chen; 2024, Hualiang Lin]

Life-course exposure to PM2.5 components and genetic susceptibility on the risk of atrial fibrillation and subsequent heart failure [2025, Hualiang Lin]

Lifestyle Factors in the Association of Shift Work With Kidney Stone Events [2025, Hualiang Lin]

Long-term exposure to ambient ozone and adult-onset asthma: A prospective cohort study [2024, Lan Chen; 2024, Hualiang Lin]

Mapping plasma metabolic profiles reflecting air pollution and their association with kidney failure: A prospective cohort study [2025, Lan Chen; 2025, Hualiang Lin]

Metabolomic profiling identifies signatures and biomarkers linking air pollution to dementia risk: A prospective cohort study [2025, Hualiang Lin]

No Associations Between Glucosamine Supplementation and Dementia or Parkinson's Disease: Findings From a Large Prospective Cohort Study [2024, Lan Chen; 2024, Hualiang Lin]

Observational and Experimental Evidence on the Interaction Between Fine Particulate Matter and Shared Genetic Variants Across Atherosclerotic Cardiovascular Disease Subtypes [2025, Lan Chen; 2025, Hualiang Lin]

Plasma metabolomic signature of healthy lifestyle, structural brain reserve and risk of dementia [2025, Hualiang Lin]

Post-cardiovascular disease healthy lifestyle, inflammation and metabolic biomarkers, and risk of dementia: a population-based longitudinal study [2025, Hualiang Lin]

Prioritizing Modifiable Risk Factors for Dementia Prevention across the Spectrum of Genetic Susceptibility: A Prospective Cohort Study [2025, Lan Chen; 2025, Hualiang Lin]

Regular use of fish oil supplements and course of cardiovascular diseases: prospective cohort study [2024, Hualiang Lin]

Sarcopenia and mild kidney dysfunction and risk of all-cause and cause-specific mortality in older adults [2024, Hualiang Lin]

The association between cytomegalovirus infection and neurodegenerative diseases: a prospective cohort using UK Biobank data [2024, Hualiang Lin]

Tobacco Smoke Exposure From Prenatal To Adolescent Periods Drives IBD Pathogenesis: Dynamic DNA Methylation Signatures Across Lifespan Stages [2026, Kun Liu]

Unraveling the genetic interplay between sleep disorders and Alzheimer's disease: From shared genes to potential therapeutic targets [2026, Hualiang Lin]

2. Please explain the key elements of your study design and the use of the available datasets that make your study an original scientific contribution	Preventing duplicate publication where published information is reorganised and republished	This study is based on the UK Biobank (UKB) prospective cohort, comprising 376,749 participants at baseline. We examined the association between residential green space (measured by NDVI at 500 m, 800 m, and 1000 m buffers) and blue space (distance to nearest water body) with incident irritable bowel syndrome (IBS) using time-to-event analysis (Cox proportional hazards models). To our knowledge, this is the first study to investigate the interaction between genetic susceptibility (polygenic risk score, PRS) and residential green/blue space in relation to IBS incidence. Additionally, we performed mediation analysis to assess the role of physical activity. The originality of this contribution lies in: (1) being the first to jointly consider genetic risk and green/blue space for IBS; (2) demonstrating that the protective effect of green space is significant only among high-genetic-risk individuals; and (3) identifying physical activity as a mediator. No previous study has reported these gene–environment interactions for IBS using large-scale repository data.
3. Please list all publications that addressed similar research questions in the same dataset and indicate where you cited them in your paper	Preventing duplicate publication where other groups have already conducted similar research in the same dataset	No publication addressing a similar research question (residential green/blue space with incident IBS) in the UK Biobank exists. Therefore, we cite no such paper in our manuscript.
4. Please explain how you addressed multiple testing through an appropriately rigorous statistical threshold and indicate this in the methods section	Preventing publication of false positive associations which are of low value unless a rigorous statistical method accounted for multiple testing	In this study, we did not apply formal multiple testing correction (e.g., Bonferroni, false discovery rate) for the following reasons: (1) the primary analyses focused on a limited number of pre-specified exposures (green space at three buffers, blue space as distance to water) and a single outcome (incident IBS); (2) secondary analyses including restricted cubic splines, mediation, and stratified analyses were considered hypothesis-generating rather than confirmatory. All reported p-values and 95% confidence intervals are presented without

		adjustment for multiple comparisons. This approach is clearly stated in the Statistical analysis subsection of the Methods.
5. Please declare to what extent have AI chatbots been used in developing your paper and to which parts of the paper did they contribute	Preventing the improper use of AI chatbots in developing the key elements of the paper and ensuring reproducibility and transparency	We used DeepSeek solely for language polishing and grammar correction. No AI chatbot contributed to study design, data analysis, results interpretation, or any substantive scientific content.

AI – artificial intelligence, JoGH – Journal of Global Health

Appendix S2.

STROBE Statement—Checklist of items that should be included in reports of ***cohort studies***

	Item No	Recommendation	Page No
**Title and abstract**	1	(*a*) Indicate the study's design with a commonly used term in the title or the abstract	1
		(*b*) Provide in the abstract an informative and balanced summary of what was done and what was found	2
**Introduction**			
Background/rationale	2	Explain the scientific background and rationale for the investigation being reported	2-3
Objectives	3	State specific objectives, including any prespecified hypotheses	3
**Methods**			
Study design	4	Present key elements of study design early in the paper	4
Setting	5	Describe the setting, locations, and relevant dates, including periods of recruitment, exposure, follow-up, and data collection	4
Participants	6	(*a*) Give the eligibility criteria, and the sources and methods of selection of participants. Describe methods of follow-up	4
		(*b*) For matched studies, give matching criteria and number of exposed and unexposed	4
Variables	7	Clearly define all outcomes, exposures, predictors, potential confounders, and effect modifiers. Give diagnostic criteria, if applicable	4-5
Data sources/ measurement	8*	For each variable of interest, give sources of data and details of methods of assessment (measurement). Describe comparability of assessment methods if there is more than one group	4-5
Bias	9	Describe any efforts to address potential sources of bias	4-5
Study size	10	Explain how the study size was arrived at	4
Quantitative variables	11	Explain how quantitative variables were handled in the analyses. If applicable, describe which groupings were chosen and why	6
Statistical methods	12	(*a*) Describe all statistical methods, including those used to control for confounding	6-7
		(*b*) Describe any methods used to examine subgroups and interactions	
		(*c*) Explain how missing data were addressed	
		(*d*) If applicable, explain how loss to follow-up was addressed	
		(*e*) Describe any sensitivity analyses	
**Results**			
Participants	13*	(a) Report numbers of individuals at each stage of study—eg numbers potentially eligible, examined for eligibility, confirmed eligible, included in the study, completing follow-up, and analysed	7-8
		(b) Give reasons for non-participation at each stage	
		(c) Consider use of a flow diagram	
Descriptive data	14*	(a) Give characteristics of study participants (eg demographic, clinical, social) and information on exposures and potential confounders	7-8
		(b) Indicate number of participants with missing data for each variable of interest	
		(c) Summarise follow-up time (eg, average and total amount)	
Outcome data	15*	Report numbers of outcome events or summary measures over time	7

Main results	16	(*a*) Give unadjusted estimates and, if applicable, confounder-adjusted estimates and their precision (eg, 95% confidence interval). Make clear which confounders were adjusted for and why they were included<br>(*b*) Report category boundaries when continuous variables were categorized<br>(*c*) If relevant, consider translating estimates of relative risk into absolute risk for a meaningful time period	8-9
Other analyses	17	Report other analyses done—eg analyses of subgroups and interactions, and sensitivity analyses	9-11
**Discussion**			
Key results	18	Summarise key results with reference to study objectives	11
Limitations	19	Discuss limitations of the study, taking into account sources of potential bias or imprecision. Discuss both direction and magnitude of any potential bias	14
Interpretation	20	Give a cautious overall interpretation of results considering objectives, limitations, multiplicity of analyses, results from similar studies, and other relevant evidence	11-13
Generalisability	21	Discuss the generalisability (external validity) of the study results	11-12
**Other information**			
Funding	22	Give the source of funding and the role of the funders for the present study and, if applicable, for the original study on which the present article is based	15

*Give information separately for exposed and unexposed groups.

Note: An Explanation and Elaboration article discusses each checklist item and gives methodological background and published examples of transparent reporting. The STROBE checklist is best used in conjunction with this article (freely available on the Web sites of PLoS Medicine at http://www.plosmedicine.org/, Annals of Internal Medicine at http://www.annals.org/, and Epidemiology at http://www.epidem.com/). Information on the STROBE Initiative is available at http://www.strobe-statement.org.

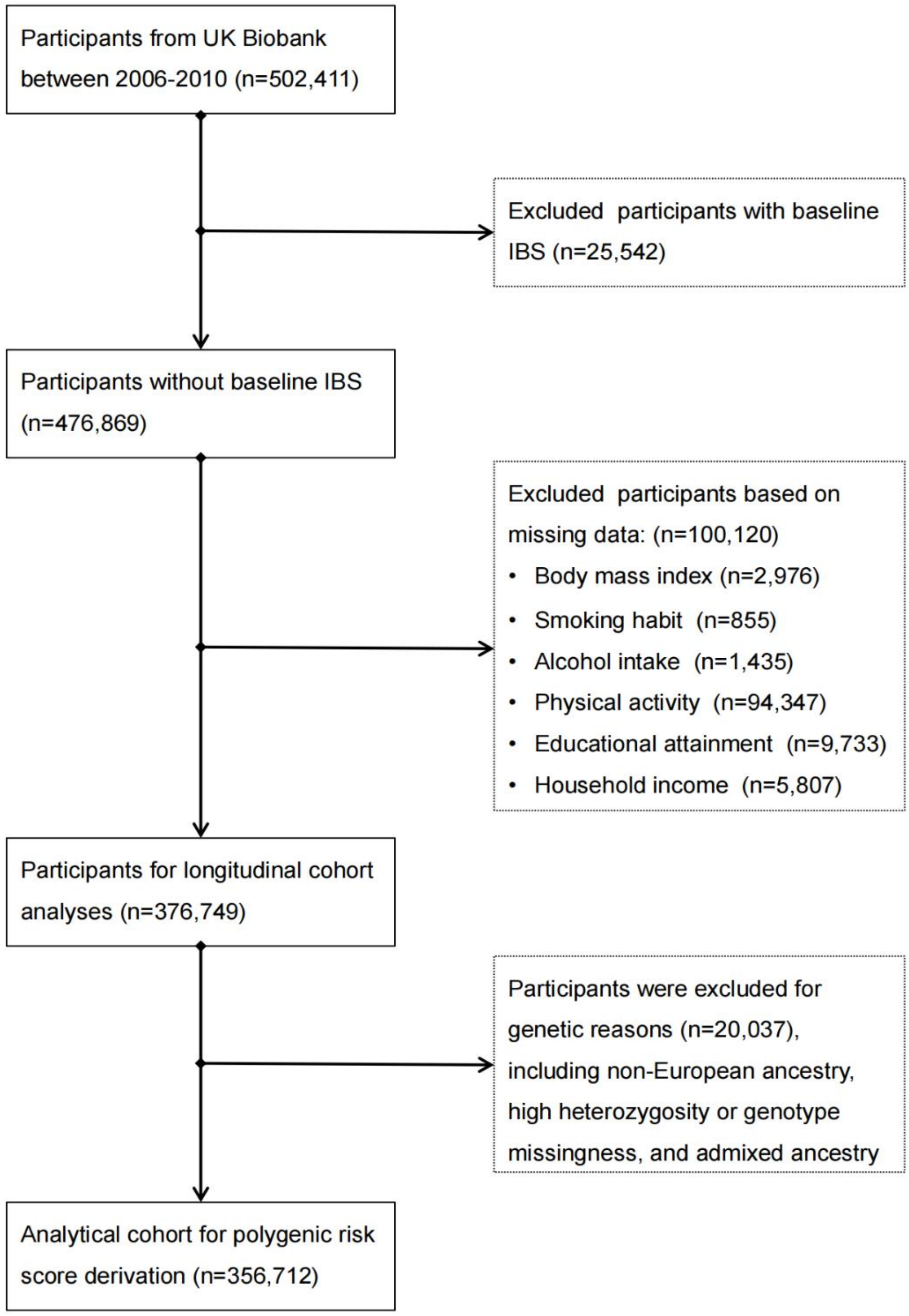

Figure S1. Flowchart of selection of study participants.

Abbreviations: IBS, irritable bowel syndrome.

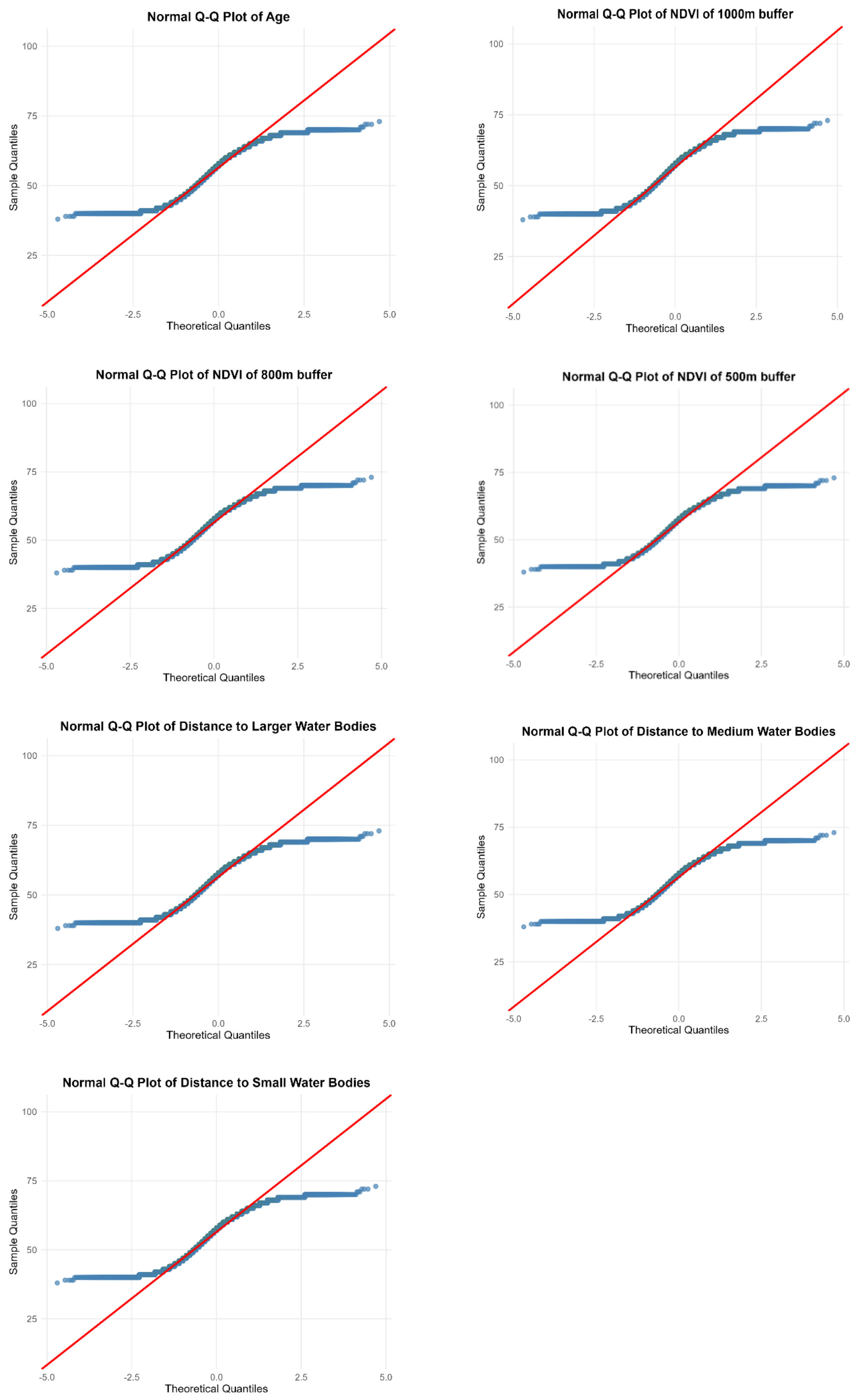

Figure S2. Normal Q-Q Plots of continuous variables.

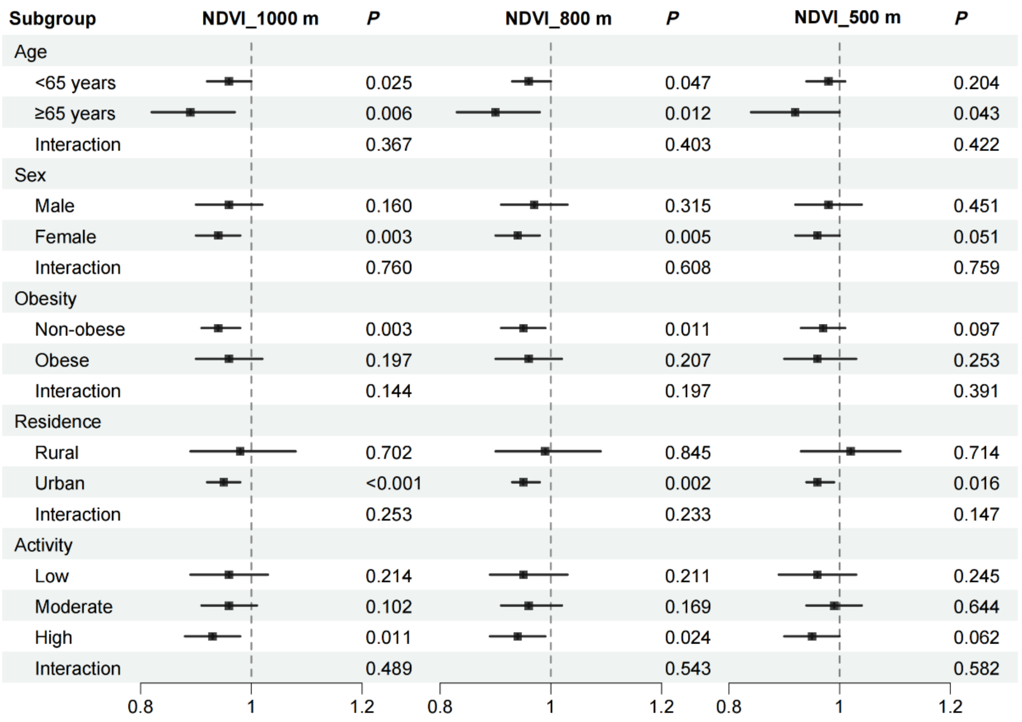

Figure S3. Forest plot of subgroup analyses the association between green space and IBS incidence. *Abbreviations:* IBS, irritable bowel syndrome; NDVI, normalized difference vegetation index.

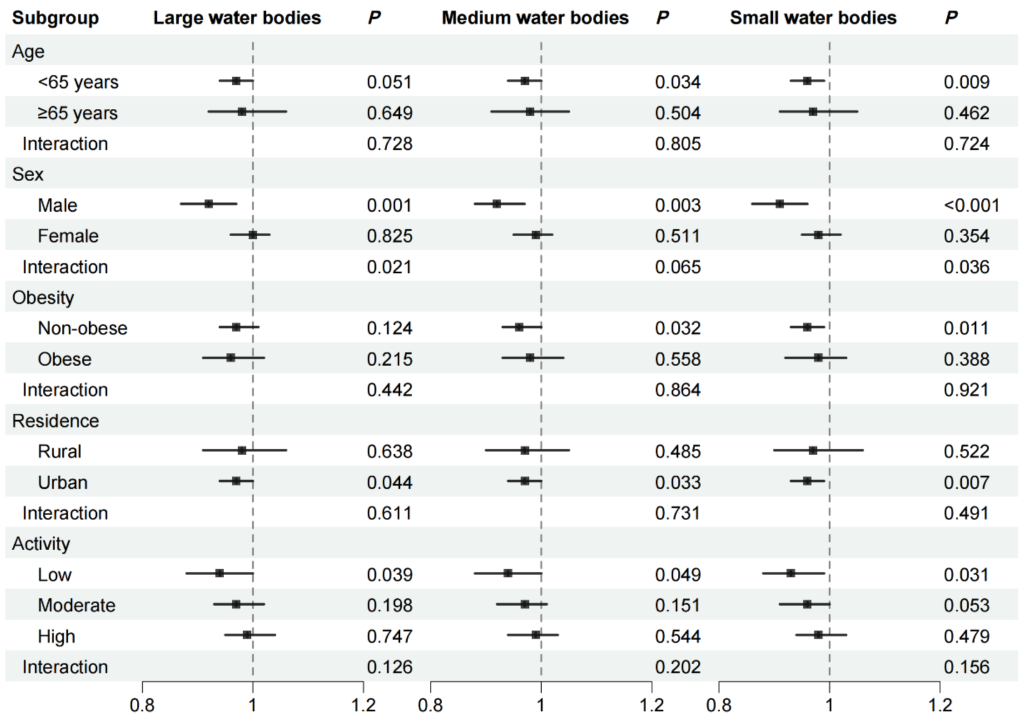

Figure S4. Forest plot of subgroup analyses the association between blue space and IBS incidence. *Abbreviations:* IBS, irritable bowel syndrome.

Table S1. Characteristics of genetic variants associated with IBS in UK Biobank

SNP	CHR	*P*-value	Allele 1	Allele 2	OR	95% CI	Frequency
rs1248825	3	7.48×10^{-15}	C	A	1.05	1.03–1.07	0.33
rs2736155	6	3.19×10^{-12}	G	C	1.05	1.02–1.07	0.48
rs10156602	9	3.04×10^{-15}	G	A	1.04	1.02–1.06	0.63
rs7106434	11	9.17×10^{-11}	C	T	1.04	1.02–1.06	0.41
rs5803650	13	6.31×10^{-14}	CT	C	1.05	1.03–1.07	0.48
rs9513519	13	2.31×10^{-10}	G	A	1.04	1.02–1.06	0.62

An allele is a variant form of a gene that occupies a specific position on a chromosome, and Allele 1 was the reference group. *Abbreviations:* CHR, chromosome; OR, odds ratio; SNP, single nucleotide polymorphism.

Table S2. Associations between blue space and the incidence of IBS

		Model 1		Model 2		Model 3	
Distance to water bodies	Cases, n (%) *	HR (95 % CI)	*P* value	HR (95 % CI)	*P* value	HR (95 % CI)	*P* value
Large water bodies							
Per IQR increment	7,091	0.96 (0.94, 0.99)	0.011	0.97 (0.94, 0.99)	0.038	0.97 (0.94, 0.99)	0.049
Q1	1,782 (25.13)	Ref	-	Ref	-	Ref	-
Q2	1,885 (26.58)	1.06 (0.99, 1.13)	0.094	1.05 (0.99, 1.12)	0.130	1.05 (0.99, 1.12)	0.128
Q3	1,791 (25.26)	1.01 (0.95, 1.08)	0.677	1.01 (0.95, 1.08)	0.677	1.02 (0.95, 1.09)	0.630
Q4	1,633 (23.03)	0.93 (0.87, 1.00)	0.041	0.94 (0.88, 1.01)	0.078	0.94 (0.88, 1.01)	0.095
Medium water bodies							
Per IQR increment	7,091	0.96 (0.93, 0.99)	0.005	0.97 (0.94, 0.99)	0.021	0.97 (0.94, 0.99)	0.028
Q1	1,769 (24.95)	Ref	-	Ref	-	Ref	-
Q2	1,877 (26.47)	1.06 (1.00, 1.13)	0.063	1.06 (0.99, 1.13)	0.094	1.06 (0.99, 1.13)	0.092
Q3	1,802 (25.41)	1.02 (0.96, 1.09)	0.552	1.02 (0.95, 1.09)	0.598	1.02 (0.96, 1.09)	0.553
Q4	1,643 (23.17)	0.94 (0.88, 1.01)	0.090	0.95 (0.89, 1.02)	0.173	0.96 (0.90, 1.02)	0.211
Small water bodies							
Per IQR increment	7,091	0.95 (0.93, 0.98)	0.002	0.96 (0.93, 0.99)	0.006	0.96 (0.93, 0.99)	0.008
Q1	1,792 (25.27)	Ref	-	Ref	-	Ref	-
Q2	1,850 (26.09)	1.04 (0.97, 1.10)	0.293	1.03 (0.96, 1.10)	0.417	1.03 (0.96, 1.10)	0.410
Q3	1,834 (25.86)	1.03 (0.97, 1.10)	0.355	1.03 (0.96, 1.09)	0.457	1.03 (0.96, 1.10)	0.429
Q4	1,615 (22.78)	0.92 (0.86, 0.98)	0.015	0.93 (0.87, 0.99)	0.027	0.93 (0.87, 0.99)	0.034

Model 1 adjusted for age, sex, and ethnicity. Model 2 additionally adjusted for residence, income, and educational level. Model 3 was further adjusted for BMI, smoking status, alcohol intake, and physical activity. * Data are presented as n (%), n represents the number of cases, with the percentage in parentheses indicating the proportion of cases within the total cases (N=7,091). Abbreviations: CI, confidence interval; HR, hazard ratio; IBS, irritable bowel syndrome; IQR, interquartile range.

Table S3. Association between blue space and IBS incidence stratified by PRS

	Low genetic risk		High genetic risk	
Distance to water bodies	HR (95 % CI)	*P* value	HR (95 % CI)	*P* value
Large water bodies				
Per IQR increment	0.97 (0.93, 1.02)	0.248	0.98 (0.94, 1.02)	0.309
Q1	Ref	-	Ref	-
Q2	1.05 (0.95, 1.15)	0.378	1.04 (0.95, 1.14)	0.353
Q3	1.01 (0.91, 1.11)	0.873	1.03 (0.94, 1.13)	0.480
Q4	0.95 (0.85, 1.05)	0.281	0.96 (0.87, 1.05)	0.346
Medium water bodies				
Per IQR increment	0.98 (0.94, 1.02)	0.282	0.97 (0.93, 1.01)	0.139
Q1	Ref	-	Ref	-
Q2	1.08 (0.98, 1.19)	0.122	1.04 (0.95, 1.13)	0.452
Q3	1.03 (0.93, 1.14)	0.560	1.03 (0.94, 1.13)	0.467
Q4	0.98 (0.89, 1.09)	0.754	0.95 (0.87, 1.05)	0.310
Small water bodies				
Per IQR increment	0.96 (0.92, 1.01)	0.105	0.97 (0.93, 1.01)	0.121
Q1	Ref	-	Ref	-
Q2	1.04 (0.95, 1.15)	0.383	1.00 (0.91, 1.09)	0.954
Q3	1.01 (0.92, 1.12)	0.796	1.05 (0.95, 1.14)	0.337
Q4	0.93 (0.84, 1.04)	0.194	0.94 (0.85, 1.03)	0.186

Cox proportional hazard model adjusted by age, sex, ethnicity, residence, income, educational level, BMI, smoking status, alcohol intake, and physical activity.

Table S4. The multiplicative interaction between the green and blue spaces

	Distance to water bodies					
	Large water bodies		Medium water bodies		Small water bodies	
Green space	HR (95 % CI)	*P*	HR (95 % CI)	*P*	HR (95 % CI)	*P*
NDVI 1,000 m buffer	0.97 (0.88,1.07)	0.493	1.00 (0.91, 1.10)	0.997	0.99 (0.90, 1.10)	0.904
NDVI 800 m buffer	0.94 (0.85, 1.04)	0.232	0.96 (0.87, 1.06)	0.401	0.95 (0.86, 1.05)	0.314
NDVI 500 m buffer	0.97 (0.88,1.07)	0.489	0.95 (0.86, 1.05)	0.283	0.93 (0.84, 1.02)	0.127

Model: Cox proportional hazard model adjusted by age, sex, ethnicity, residence, income, educational level, BMI, smoking status, alcohol intake, and physical activity.

Abbreviations: CI, confidence interval; HR, hazard ratio; NDVI, normalized difference vegetation index.

Table S5. Associations of green space and IBS incidence (restricting IBS analyses to non-movers during follow-up)

	Model 1		Model 2		Model 3	
	HR (95 % CI)	*P*	HR (95 % CI)	*P*	HR (95 % CI)	*P*
NDVI 1,000 m buffer						
Per IQR increment	0.98 (0.94, 1.01)	0.166	0.95 (0.91, 0.99)	0.018	0.94 (0.90, 0.98)	0.008
Q1	Ref	-	Ref	-	Ref	-
Q2	0.98 (0.90, 1.06)	0.553	0.94 (0.87, 1.02)	0.163	0.94 (0.87, 1.02)	0.147
Q3	0.93 (0.86, 1.00)	0.064	0.89 (0.81, 0.97)	0.008	0.88 (0.81, 0.96)	0.005
Q4	0.96 (0.88, 1.03)	0.262	0.92 (0.85, 1.01)	0.080	0.91 (0.83, 1.00)	0.040
NDVI 800 m buffer						
Per IQR increment	0.98 (0.95, 1.02)	0.312	0.96 (0.92, 1.00)	0.056	0.95 (0.91, 1.00)	0.029
Q1	Ref	-	Ref	-	Ref	-
Q2	1.05 (0.98, 1.14)	0.186	1.02 (0.94, 1.11)	0.625	1.02 (0.94, 1.11)	0.694
Q3	0.96 (0.88, 1.03)	0.264	0.92 (0.84, 1.01)	0.078	0.92 (0.84, 1.00)	0.052
Q4	0.98 (0.91, 1.07)	0.705	0.96 (0.88, 1.05)	0.381	0.95 (0.87, 1.04)	0.241
NDVI 500 m buffer						
Per IQR increment	0.99 (0.96, 1.03)	0.674	0.98 (0.94, 1.02)	0.264	0.97 (0.93, 1.01)	0.178
Q1	Ref	-	Ref	-	Ref	-
Q2	1.04 (0.96, 1.12)	0.355	1.01 (0.93, 1.10)	0.795	1.01 (0.93, 1.10)	0.851
Q3	1.00 (0.93, 1.07)	0.993	0.98 (0.91, 1.06)	0.587	0.97 (0.90, 1.05)	0.448
Q4	0.84 (0.63, 1.10)	0.203	0.82 (0.62, 1.09)	0.176	0.82 (0.62, 1.08)	0.163

Model 1: Cox proportional hazard model adjusted by age, sex and ethnicity; Model 2: further adjusted by residence, income and educational level.
Model 3: further adjusted by BMI, smoking status, alcohol intake, and physical activity. *Abbreviations:* CI, confidence interval; HR, hazard ratio; IBS, irritable bowel syndrome; IQR, interquartile range; NDVI, normalized difference vegetation index.

Table S6. Associations of green space and IBS incidence (Multiple imputation of missing covariate data)

	Model 1		Model 2		Model 3	
Green space	HR (95 % CI)	*P*	HR (95 % CI)	*P*	HR (95 % CI)	*P*
NDVI 1,000 m buffer						
Per IQR increment	0.98 (0.95, 1.01)	0.105	0.95 (0.92, 0.98)	0.004	0.95 (0.91, 0.98)	0.001
Q1	Ref	-	Ref	-	Ref	-
Q2	0.99 (0.93, 1.06)	0.737	0.96 (0.90, 1.03)	0.250	0.96 (0.89, 1.03)	0.221
Q3	0.96 (0.90, 1.02)	0.198	0.92 (0.85, 0.99)	0.019	0.91 (0.85, 0.98)	0.012
Q4	0.94 (0.88, 1.00)	0.069	0.91 (0.84, 0.98)	0.010	0.90 (0.83, 0.96)	0.003
NDVI 800 m buffer						
Per IQR increment	0.98 (0.95, 1.01)	0.179	0.96 (0.92, 0.99)	0.011	0.95 (0.92, 0.98)	0.004
Q1	Ref	-	Ref	-	Ref	-
Q2	1.04 (0.97, 1.11)	0.247	1.01 (0.94, 1.08)	0.834	1.00 (0.94, 1.07)	0.926
Q3	0.97 (0.91, 1.04)	0.387	0.93 (0.87, 1.00)	0.065	0.93 (0.86, 0.99)	0.040
Q4	0.98 (0.92, 1.05)	0.567	0.95 (0.88, 1.02)	0.185	0.94 (0.87, 1.01)	0.099
NDVI 500 m buffer						
Per IQR increment	0.99 (0.96, 1.02)	0.478	0.97 (0.94, 1.00)	0.081	0.97 (0.93, 0.99)	0.045
Q1	Ref	-	Ref	-	Ref	-
Q2	1.05 (0.99, 1.12)	0.121	1.03 (0.96, 1.10)	0.480	1.02 (0.95, 1.09)	0.539
Q3	1.01 (0.95, 1.07)	0.793	0.98 (0.92, 1.05)	0.551	0.97 (0.91, 1.04)	0.392
Q4	0.84 (0.68, 1.05)	0.130	0.83 (0.67, 1.04)	0.102	0.83 (0.66, 1.03)	0.092

Model 1: Cox proportional hazard model adjusted by age, sex and ethnicity; Model 2: further adjusted by residence, income and educational level.
Model 3: further adjusted by BMI, smoking status, alcohol intake, and physical activity. *Abbreviations:* CI, confidence interval; HR, hazard ratio; IBS, irritable bowel syndrome; IQR, interquartile range; NDVI, normalized difference vegetation index.

Table S7. Associations of green space and IBS incidence (adjusting for additional clinical covariates)

	Model 1		Model 2		Model 3	
Green space	HR (95 % CI)	*P*	HR (95 % CI)	*P*	HR (95 % CI)	*P*
NDVI 1,000 m buffer						
Per IQR increment	0.98 (0.95, 1.00)	0.088	0.95 (0.92, 0.98)	0.003	0.95 (0.91, 0.98)	0.001
Q1	Ref	-	Ref	-	Ref	-
Q2	0.99 (0.93, 1.05)	0.720	0.96 (0.90, 1.03)	0.247	0.96 (0.89, 1.03)	0.219
Q3	0.96 (0.90, 1.02)	0.181	0.92 (0.85, 0.99)	0.018	0.91 (0.85, 0.98)	0.012
Q4	0.94 (0.88, 1.00)	0.058	0.91 (0.84, 0.97)	0.008	0.90 (0.83, 0.96)	0.003
NDVI 800 m buffer						
Per IQR increment	0.98 (0.95, 1.01)	0.154	0.96 (0.92, 0.99)	0.009	0.95 (0.92, 0.98)	0.004
Q1	Ref	-	Ref	-	Ref	-
Q2	1.04 (0.97, 1.11)	0.258	1.01 (0.94, 1.08)	0.839	1.00 (0.94, 1.07)	0.929
Q3	0.97 (0.91, 1.04)	0.359	0.93 (0.87, 1.00)	0.062	0.93 (0.86, 0.99)	0.039
Q4	0.98 (0.91, 1.05)	0.513	0.95 (0.88, 1.02)	0.167	0.94 (0.87, 1.01)	0.092
NDVI 500 m buffer						
Per IQR increment	0.99 (0.96, 1.02)	0.433	0.97 (0.94, 1.00)	0.074	0.97 (0.93, 0.99)	0.043
Q1	Ref	-	Ref	-	Ref	-
Q2	1.05 (0.99, 1.12)	0.127	1.02 (0.96, 1.10)	0.482	1.02 (0.95, 1.09)	0.539
Q3	1.01 (0.95, 1.07)	0.850	0.98 (0.92, 1.04)	0.526	0.97 (0.91, 1.04)	0.381
Q4	0.84 (0.67, 1.05)	0.123	0.83 (0.66, 1.04)	0.098	0.82 (0.66, 1.03)	0.088

Model 1: Cox proportional hazard model adjusted by age, sex, ethnicity, hypertension and diabetes; Model 2: further adjusted by residence, income, educational level, hypertension and diabetes. Model 3: further adjusted by BMI, smoking status, alcohol intake, physical activity, hypertension and diabetes. *Abbreviations:* CI, confidence interval; HR, hazard ratio; IBS, irritable bowel syndrome; IQR, interquartile range; NDVI, normalized difference vegetation index.

Table S8. Associations of blue space and IBS incidence (restricting IBS analyses to non-movers during follow-up)

	Model 1		Model 2		Model 3	
Distance to water bodies	HR (95 % CI)	*P*	HR (95 % CI)	*P*	HR (95 % CI)	*P*
Large water bodies						
Per IQR increment	0.98 (0.94, 1.01)	0.147	0.98 (0.95, 1.01)	0.259	0.98 (0.95, 1.02)	0.297
Q1	Ref	-	Ref	-	Ref	-
Q2	1.05 (0.97, 1.13)	0.230	1.04 (0.96, 1.13)	0.294	1.04 (0.97, 1.13)	0.289
Q3	1.06 (0.98, 1.14)	0.148	1.06 (0.98, 1.14)	0.166	1.06 (0.98, 1.14)	0.146
Q4	0.95 (0.88, 1.03)	0.193	0.95 (0.88, 1.03)	0.246	0.96 (0.88, 1.04)	0.275
Medium water bodies						
Per IQR increment	0.97 (0.94, 1.01)	0.110	0.98 (0.94, 1.01)	0.201	0.98 (0.95, 1.01)	0.244
Q1	Ref	-	Ref	-	Ref	-
Q2	1.06 (0.98, 1.14)	0.147	1.05 (0.97, 1.14)	0.194	1.05 (0.98, 1.14)	0.182
Q3	1.06 (0.98, 1.15)	0.119	1.06 (0.98, 1.15)	0.141	1.06 (0.98, 1.15)	0.124
Q4	0.96 (0.89, 1.04)	0.329	0.97 (0.89, 1.05)	0.445	0.97 (0.90, 1.05)	0.504
Small water bodies						
Per IQR increment	0.96 (0.93, 0.99)	0.023	0.97 (0.93, 0.99)	0.046	0.97 (0.93, 1.00)	0.057
Q1	Ref	-	Ref	-	Ref	-
Q2	1.00 (0.93, 1.08)	0.958	1.00 (0.92, 1.08)	0.904	1.00 (0.92, 1.08)	0.926
Q3	1.04 (0.96, 1.12)	0.368	1.03 (0.95, 1.11)	0.459	1.03 (0.96, 1.11)	0.424
Q4	0.92 (0.85, 0.99)	0.036	0.92 (0.85, 1.00)	0.052	0.93 (0.85, 1.00)	0.059

Model 1: Cox proportional hazard model adjusted by age, sex and ethnicity; Model 2: further adjusted by residence, income and educational level.
Model 3: further adjusted by BMI, smoking status, alcohol intake, and physical activity. *Abbreviations*: CI, confidence interval; HR, hazard ratio; IBS, irritable bowel syndrome; IQR, interquartile range.

Table S9. Associations of blue space and IBS incidence (Multiple imputation of missing covariate data)

	Model 1		Model 2		Model 3	
Distance to water bodies	HR (95 % CI)	*P*	HR (95 % CI)	*P*	HR (95 % CI)	*P*
Large water bodies						
Per IQR increment	0.96 (0.93, 0.98)	<0.001	0.97 (0.94, 0.99)	0.007	0.97 (0.94, 0.99)	0.011
Q1	Ref	-	Ref	-	Ref	-
Q2	1.07 (1.01, 1.13)	0.026	1.06 (1.00, 1.12)	0.038	1.06 (1.00, 1.12)	0.034
Q3	0.98 (0.93, 1.04)	0.561	0.98 (0.93, 1.04)	0.596	0.99 (0.93, 1.04)	0.646
Q4	0.93 (0.88, 0.99)	0.021	0.95 (0.89, 1.00)	0.066	0.95 (0.90, 1.01)	0.088
Medium water bodies						
Per IQR increment	0.95 (0.93, 0.98)	<0.001	0.96 (0.94, 0.99)	0.002	0.96 (0.94, 0.99)	0.004
Q1	Ref	-	Ref	-	Ref	-
Q2	1.06 (1.01, 1.13)	0.031	1.06 (1.00, 1.12)	0.045	1.06 (1.00, 1.12)	0.040
Q3	1.00 (0.95, 1.06)	0.888	1.00 (0.95, 1.06)	0.909	1.01 (0.95, 1.06)	0.842
Q4	0.93 (0.88, 0.99)	0.015	0.94 (0.89, 1.00)	0.055	0.95 (0.89, 1.01)	0.076
Small water bodies						
Per IQR increment	0.95 (0.93, 0.97)	<0.001	0.96 (0.93, 0.98)	<0.001	0.96 (0.93, 0.98)	0.001
Q1	Ref	-	Ref	-	Ref	-
Q2	1.04 (0.99, 1.10)	0.130	1.04 (0.98, 1.10)	0.188	1.04 (0.98, 1.10)	0.182
Q3	1.01 (0.96, 1.07)	0.609	1.01 (0.96, 1.07)	0.696	1.01 (0.96, 1.07)	0.648
Q4	0.91 (0.86, 0.96)	0.002	0.92 (0.87, 0.98)	0.005	0.92 (0.87, 0.98)	0.007

Model 1: Cox proportional hazard model adjusted by age, sex and ethnicity; Model 2: further adjusted by residence, income and educational level.
Model 3: further adjusted by BMI, smoking status, alcohol intake, and physical activity. *Abbreviations*: CI, confidence interval; HR, hazard ratio; IBS, irritable bowel syndrome; IQR, interquartile range.

Table S10. Associations of blue space and IBS incidence (adjusting for additional clinical covariates)

	Model 1		Model 2		Model 3	
Distance to water bodies	HR (95 % CI)	*P*	HR (95 % CI)	*P*	HR (95 % CI)	*P*
Large water bodies						
Per IQR increment	0.96 (0.94, 0.99)	0.012	0.97 (0.94, 0.99)	0.040	0.97 (0.94, 1.00)	0.052
Q1	Ref	-	Ref	-	Ref	-
Q2	1.06 (0.99, 1.13)	0.095	1.05 (0.99, 1.12)	0.132	1.05 (0.99, 1.12)	0.129
Q3	1.01 (0.95, 1.08)	0.671	1.01 (0.95, 1.08)	0.675	1.02 (0.95, 1.09)	0.630
Q4	0.93 (0.87, 0.99)	0.045	0.94 (0.88, 1.01)	0.082	0.94 (0.88, 1.01)	0.099
Medium water bodies						
Per IQR increment	0.96 (0.93, 0.99)	0.006	0.97 (0.94, 0.99)	0.021	0.97 (0.94,0.99)	0.029
Q1	Ref	-	Ref	-	Ref	-
Q2	1.06 (1.00, 1.13)	0.065	1.06 (0.99, 1.13)	0.095	1.06 (0.99, 1.13)	0.093
Q3	1.02 (0.96, 1.09)	0.545	1.02 (0.95, 1.09)	0.595	1.02 (0.96, 1.09)	0.554
Q4	0.94 (0.88, 1.01)	0.097	0.95 (0.89, 1.02)	0.179	0.96 (0.90, 1.03)	0.215
Small water bodies						
Per IQR increment	0.96 (0.93, 0.98)	0.002	0.96 (0.93, 0.99)	0.006	0.96 (0.93, 0.99)	0.008
Q1	Ref	-	Ref	-	Ref	-
Q2	1.03 (0.97, 1.10)	0.301	1.03 (0.96, 1.10)	0.423	1.03 (0.96, 1.10)	0.413
Q3	1.03 (0.97, 1.10)	0.353	1.03 (0.96, 1.09)	0.457	1.03 (0.96, 1.10)	0.430
Q4	0.92 (0.86, 0.99)	0.017	0.93 (0.87, 0.99)	0.028	0.93 (0.87, 0.99)	0.035

Model 1: Cox proportional hazard model adjusted by age, sex, ethnicity, hypertension and diabetes; Model 2: further adjusted by residence, income, educational level, hypertension and diabetes. Model 3: further adjusted by BMI, smoking status, alcohol intake, physical activity, hypertension and diabetes. *Abbreviations*: CI, confidence interval; HR, hazard ratio; IBS, irritable bowel syndrome; IQR, interquartile range.
